# A Fluorescent Tile DNA Diagnocode System for *In Situ* Rapid and Selective Diagnosis of Cytosolic RNA Cancer Markers

**DOI:** 10.1038/srep18497

**Published:** 2015-12-18

**Authors:** Kyung Soo Park, Seung Won Shin, Min Su Jang, Woojung Shin, Kisuk Yang, Junhong Min, Seung-Woo Cho, Byung-Keun Oh, Jong Wook Bae, Sunghwan Jung, Jeong-Woo Choi, Soong Ho Um

**Affiliations:** 1 School of Chemical Engineering, Sungkyunkwan University, Suwon, Gyeonggi-do, 440-746, South Korea; 2 SKKU Advanced Institute of Nanotechnology (SAINT), Sungkyunkwan University, Suwon, Gyeonggi-do, 440-746, South Korea; 3 Department of Biotechnology, Yonsei University, Seoul, 120-749, South Korea; 4 School of Integrative Engineering, Chung-Ang University, Seoul, 156-756, South Korea; 5 Department of Chemical and Biomolecular Engineering, Sogang University, Seoul, 121-742, South Korea; 6 Department of Engineering Science and Mechanics, Virginia Tech, Blacksburg, VA 24061, USA

## Abstract

Accurate cancer diagnosis often requires extraction and purification of genetic materials from cells, and sophisticated instrumentations that follow. Otherwise in order to directly treat the diagnostic materials to cells, multiple steps to optimize dose concentration and treatment time are necessary due to diversity in cellular behaviors. These processes may offer high precision but hinder fast analysis of cancer, especially in clinical situations that need rapid detection and characterization of cancer. Here we present a novel fluorescent tile DNA nanostructure delivered to cancer cytosol by employing nanoparticle technology. Its structural anisotropicity offers easy manipulation for multifunctionalities, enabling the novel DNA nanostructure to detect intracellular cancer RNA markers with high specificity within 30 minutes post treatment, while the nanoparticle property bypasses the requirement of treatment optimization, effectively reducing the complexity of applying the system for cancer diagnosis. Altogether, the system offers a precise and rapid detection of cancer, suggesting the future use in the clinical fields.

A myriad of nanotechnological tool kits for cancer diagnosis have been developed in the recent decades^1^,^2^,^3^,^4^. In particular, DNA as a basic material for cancer research is gaining much attention due to its accommodation for precise enzymatic manipulation, intrinsic biocompatibility, and high target specificity^5^,^6^,^7^. Timely cancer diagnosis is necessary to realize an efficacious process to optimize the essential conditions respective to the chosen cancer species. However, most of the conventional systems for cancer diagnosis incorporate only a single reporting module which often make preconditioning process complex, delaying diagnostic procedures or even leading to biased or incorrect results. That is, the treatment duration with diagnostic particles needs to be controlled with high precision, in addition to the need for appropriate selection of modular component depending on the nature of cell of interest^8^,^9^,^10^,^11^,^12^,^13^. Meanwhile, DNA is subject to several limitations in the cytosolic environment, such as fast enzymatic degradation^14^. Here we describe a fluorescent tile DNA-based rapid and real-time cancer diagnostic tool kit. Due to its physicochemical characteristics and the rapid detection of cytosolic RNA, the system is capable of diagnosing cancer without the need for meticulous consideration of inherent cellular features.

## Results

### Synthesis of Fluorescent Tile DNA Diagnocode

**Figure 1:**
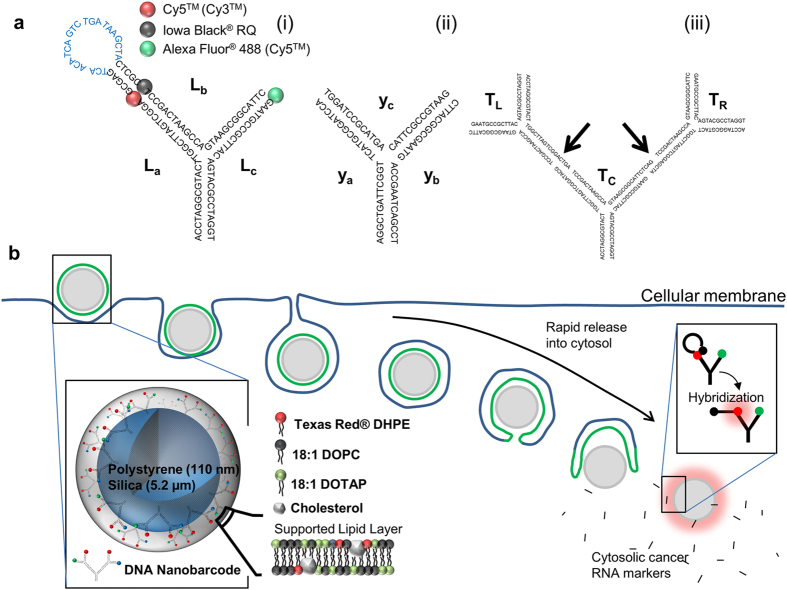
Structural design and evaluation of fluorescent tile DNA constructs. (**a**) Schematic drawings of DNA nanostructures. From left to right: Looped-DNA (L-DNA) (i), Y-shaped DNA (Y-DNA) (ii) and tree-shaped DNA (T-DNA) (iii). All DNA structures were assembled from the predesigned oligonucleotides listed in Table S1. Each DNA construct contained expected color codes and structures. For example, the one black and two colored globoids in L-DNA (i) represent a quencher and two fluorophores with different functionalities, respectively. Also, the portion designed to detect and hybridize with cytosolic cancer RNA markers is indicated with blue lettered sequence. Y-DNA (ii) was a mixture of three different structures (e.g., y_a_, y_b_ and y_c_), while T-DNA (iii) was composed of three different oligonucleotides, T_C_, T_L_ and T_R_, and was synthesized using the same method as (i). Note that T_C_ crosslinked T_L_ and T_R_ (indicated by arrows) through overhang hybridization and T4 enzymatic ligation. (**b**) Schematic drawing of cancer diagnosis process. As a core materials, the micro- or nano-scaled bead can be used for anchoring DNA nanostructures. Fused lipid layer was composed of varying mass ratios of DOTAP, DOPC, cholesterol, and if necessary, Texas Red^®^ DHPE. Once the nano-scaled particle enters into cytosol, it rapidly exposes L-DNA to the cytosolic environment. Upon the exposure, the L-DNA hybridizes with cytosolic cancer RNA markers to emit fluorescence.

We first synthesized a new tile DNA nanostructure, termed as looped DNA (L-DNA), having a hybrid form of DNA beacon^15^ and tree-shaped DNA structure (T-DNA)^16^,^17^ (Fig. 1a; note here that Y-shaped DNA (Y-DNA) was used as proof-of-concept, where T-DNA is a build-up of Y-DNAs). During the DNA nanostructure synthesis, external temperature is controlled to anneal oligonucleotides into designed nanostructures. Temperature profile used to create tile DNA nanostructures is shown in Fig. S1. The formation of DNA nanostructures is confirmed with aragose gel electrophoresis (Fig. S2). The precise complementary binding nature of oligonucloetides allows the formation of DNA nanostructures in a predetermined manner. In case of Y-DNA, three different moieties can be attached to each arm as designed. This anisotropism of Y-DNA and T-DNA can be applied to multi-coding system. Here we demonstrated the application of tile DNA nanostructures as a platform for a multi-color fluorscence coding system by attaching various fluorescent dyes, including 6-FAM^TM^, TEX^TM^615, Cy3^TM^, Cy5^TM^ and Alexa Fluor^®^ 488, to the arms of the Y- and T-DNA nanostructures. Various fluorescent colors could be expressed depending on the combination of fluorescent dyes, while by keeping one of the arms biotinylated, the DNA nanostructures could be conjugated to the surface of streptavidin coated nanobeads for signal concentration (Fig. S3, Supplementary Information). The use of DNA nanostructures as a platform to combine fluorophores turned out to better express distinct fluorescent colors compared to demonstrating them by physical mixing separate fluorophores (Fig. S4, Supplementary Information). Owing to the distinctness of expressed colors, different color codes could be preciesly distinguished (Fig. S5, Supplementary Information). Moreover, the signal intensity could be easily adjusted by controlling the amount of tile DNA conjugated to the bead surface (Fig. S6, Supplementary Information).

Furthermore, fluorescence-quencher interactive loop-arms were integrated into the T-DNA to create L-DNA capable of measuring the fluorescent signal changes induced by Förster Resonance Energy Transfer (FRET)^18^. Owing to its anisotropic nature, the L-DNA included Iowa Black^®^ RQ as a quencher, a Cy5^TM^ fluorophore indicating the number of L-DNAs responding to a cytosolic RNA marker, and Alexa Fluor^®^ 488 as a quantitative indicator for the total number of existing L-DNA (Fig. 1a,i). The anisotropic structure of L-DNA realizes double reporting system that is capable of quantifying cytosolic cancer RNA markers and enumerating a total number of systemic molecules at the same time. By evaluating the ratio of the two, this system can evade the need for a meticulous consideration for inherent features that cells have. Additionally, DNA nanostructures are conjugated to nano-sized solid beads enclosed in lipid layers in order to facilitate the rapid exposure of DNA nanostructures in cytosolic environments upon entrance into the cell (Fig. 1b). This system enabled rapid detection of the cytosolic cancer RNA markers before significant enzymatic degradation of the DNA nanostructures.

### Functional Analysis of L-DNA Diagnocode System and Confirmation of Rapid Release to Cytosol

To enable our system in cancer diagnosis and to identify and quantify cancer RNA markers in the cytosol, L-DNA with multi-functional modules was designed to capture the emitted signals of target cancer-specific RNAs and simultaneously monitor fluorescence variation for rapid quantification. Once the L-DNA randomly encountered complementary RNA, the loop structure deformed and opened up into linear DNA, enhancing its fluorescence signal proportional to the level of the matching RNA (Fig. S7, Supplementary Information). Quantitatively estimating the ratio between Cy5^TM^ and Alexa Fluor^®^ 488 allows the concentration of target RNA within the solution to be measured, which can be used to predict the presence and stage of cancer in the cells.

**Figure 2:**
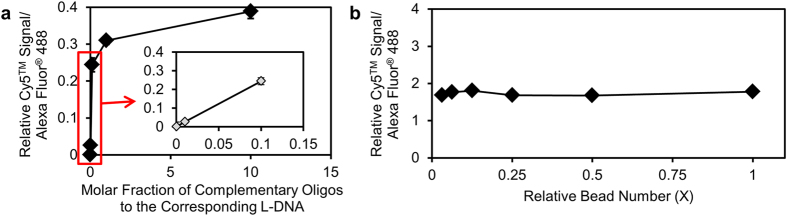
Real-time code signal monitoring of L-DNA diagnocode in solution through FRET. (**a**) L-DNA-armed diagnocodes reacted selectively with varying concentrations of complementary oligonucleotides. The relative Cy5^TM^ to Alexa Fluor^®^ 488 signal ratio increased with addition of higher concentrations of the oligonucleotide complementary to the loop sequence of the L-DNA. The trend line of the initial three points had a determination coefficient value (R^2^) near 1 (see the red box and also inset graph), indicating that this system was highly sensitive to the added target oligonucleotides, even at early time points. Without sacrificing accuracy, the sensitivity limit was increased to approximately 6.64 × 10^2^ yoctomole of the complementary oligonucleotides per diagnocode. (**b**) Varying numbers of diagnocodes were added to a fixed concentration of complementary oligonucleotides. Pairwise comparisons of relative Cy5^TM^ to Alexa Fluor^®^ 488 signal ratios at different relative bead (diagnocode) numbers revealed that the signal ratio was independent of the number of diagnocodes, but heavily dependent upon the mass of complementary oligonucleotides in solution (ANOVA test, p = 0.133). All data were obtained from triplicate experiments.

To evaluate functionality for cancer RNA marker detection, L-DNA were conjugated onto 110-nm-sized polystyrene beads, which is termed as a diagnocode. By attaching the L-DNA on polystyrene bead, local condensation of DNA nanostrucure can be obtained, and provide concentration of fluorescence signals that allows the feasible observation of the signals. Diagnocode consisted with L-DNA tested for the ability to hybridize with different concentrations of an oligonucleotide sequence from the breast cancer-specific enhancer zeste homolog 2 (EZH2, CAGATTTCTTCCCAGTCTGGC). After several hours of mixing, L-DNA was exposed to declining temperatures (Fig. S8, Supplementary Information). Some noise signals were induced by thermodynamic instability of the loop structure near its melting temperature (around 35 °C), and so the test solution was incubated at 4 °C. Interestingly, the relative signal ratios of Cy5^TM^ and Alexa Fluor^®^ 488 correlated proportionally with the increased concentration of the complementary oligonucleotide (Fig. 2a). The system was sensitive down to detection of as little as 664 yoctomole of complementary oligonucleotide fragments per L-DNA-immobilized diagnocode. However, once the concentration of the complementary oligonucleotide was considerably higher, the relative signal ratios remained constant irrespective of the number of particles, where none of the pairwise comparisons revealed a significant difference among the signal ratios (Fig. 2b, ANOVA test, p = 0.133). This finding indicates that the number of complementary oligonucleotides may be a principal marker of the degree of cancer progression. The concept of dividing induced signal with reference signal, named as relative signal, has a theoretical meaning of detecting signal without considering the total amount of diagnocodes. If we consider the complementary hydridization as a reaction process, reaction can be demonstrated in each equation like below;


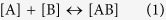


Where A and B represent complementary oligonucleotide and diagnocode, respectively, and AB is their hybridized structure. The fluorescence signal would be proportional to the concentration of the molecule that emits the fluorescence. Therefore, the relative signal ratio can be expressed by dividing the concentration of AB complex, [AB], by that of total B, [B_0_]. Thus,


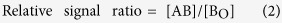


From the overall reaction equation, the dissociation constant (K_d_) can be defined as:


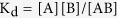


Initial concentration of diagnocode can be expressed as,


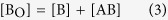


Thus, by plugging [B_0_] to the dissociation constant equation,


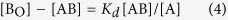



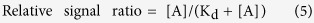


As can be confirmed from the above theoretical calculation, the relative signal is dependent only on [A] which is consistent with the data shown in Fig. 2. The relative signal ratio increases following the concentration of complementary oligonucleotide (Fig. 2a), but no significant change is observed by the change in diagnocode concentration (Fig. 2b).

**Figure 3:**
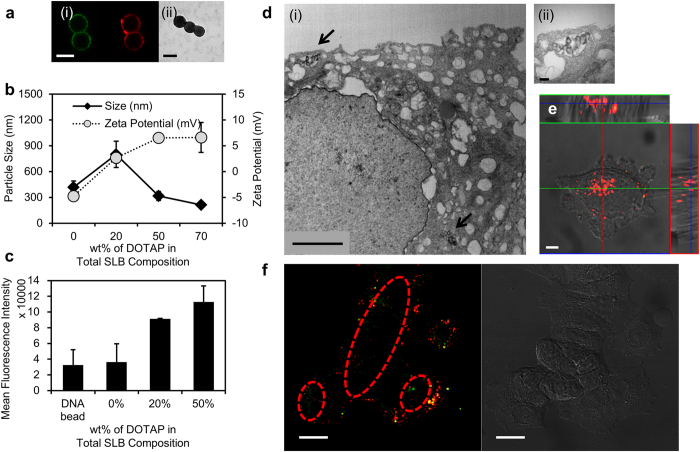
Physicochemical evaluation and characterization of entrance into cytosol of the diagnocodes using Y-DNA. (**a**) (i) shows the red-color-labeled lipid layer and green-color-labeled FCP. Diagnocodes about 100 nm in diameter are also shown in TEM (ii). (**b**) DLS analysis of diagnocodes’ size and surface charge. The size and surface charge changed with the lipid composition, mainly due to the electrostatics of the lipid. (**c**) Flow cytometry analysis of the interaction between lipid compositions on diagnocodes and a model cancer cell (PC-3). Increased DOTAP as a portion of total lipid composition led to enhanced cellular uptake. Each experiment was run in triplicate. (**d**) Evidence of the cytosolic entrance of diagnocodes (arrows) based on the size of diagnocodes as confirmed by TEM and (**e**) confocal microscope. (**f** ) Confocal imaging analysis after separation of lipid layers from diagnocodes. The image was taken 30 minutres post-treatment of diagnocodes to breast cancer cells (MCF-7). Diagnocodes were labeled with green fluorescent dye (6-FAM^TM^) and covered with liposomes labeled with red fluorescent dye (Texas Red^®^ DHPE). Yellow fluorescence indicates merge of the two fluorescence colors, which are mainly placed in extra cellular matrix. Interestingly, green fluorescence from diagnocodes was observed separate from the lipid layer (red dotted circles), indicating that the diagnocode and the liposome may have separated from each other within 30 minutes of entry into the cytosol. The scale bars are 5 μm for a(i), 100 nm for a(ii), 2 μm for d(i), 200 nm for d(ii), 5 μm for e and 20 μm for f.

To explore fluorescence-color-tagged DNA codes in a cellular environment, diagnocodes were enclosed in a lipid vesicle via fusion technology^19^,^20^. Lipid layer coating of diagnocodes through fusion technology provide improved biocompatibility, and effective surface coating. In order to verify the lipid layer coverage at the surface of the diagnocodes, two different lipid components, 1,2-dioleoyl-3-trimethylammonium-propane (DOTAP) and 1,2-dioleoyl-sn-glycero-3-phosphocholine (DOPC), were tested at mass compositions ranging from 0 to 70 wt% with 30 wt% of cholesterol. Liposomes of various lipid compositions and the resulting synthetic lipid-supported diagnocode were characterized by confocal microscopy, dynamic light scattering (DLS), and transmission electron microscopy (TEM) (Fig. 3a–c and Fig. S9, Supplementary Information). For confocal microscopy imaging of lipid fusion process, 5-μm-sized-silica bead was used. The lipid layer surrounding the diagnocode was visualized by labeling the lipids with Texas Red^®^ DHPE (Red) and the DNA structure with FITC (Green) (Fig. 3a,i). The resulting diagnocode was observable using TEM (Fig. 3a,ii). The size and surface charge of the diagnocodes varied after fusion. Size gradually decreased in proportion to the change in surface charge (Fig. 3b), due to charge repulsions among the formed particles^21^. The interaction between the particles and cells changed according to the composition of the lipid (Fig. 3c).

Cytosolic entrance of the cancer diagnocodes was visualized by a variety of microscopic tools including confocal microscopy and TEM (Fig. 3d–f). Red color-coded cancer diagnocodes were observed inside of target cells using confocal microscopy, and TEM images supported the presence of cancer diagnocodes inside the cell. In order to determine the proper time to remove the lipid layer of the diagnocodes for exposure of L-DNA to the cytosolic environment, the green fluorescence-exhibiting diagnocodes covered with a cationic lipid layer (DOTAP) labeled with Texas Red^®^ DHPE (red fluorescence) were applied to breast cancer cells (MCF-7) for 30 minutes. The cells were washed and incubated at 37 °C for varying time periods (0, 0.5, 1, 2 and 4 hrs). Notably, even the case with no additional incubation exhibited green fluorescence within the cytosolic areas, indicating that the DNA was exposed to the cytosol within 30 minutes of treatment when the lipid layer was removed (Fig. 3f). The other cases with different incubation times exhibited similar behavior, while no fluorescence signal was detectable if no diagnocode was applied (data not shown). To use L-DNA for quantifying a cytosolic RNA overflow in cancer cells, L-DNA containing a hairpin-looped DNA structure with a sequence complementary to EZH2 mRNA was conjugated to 110-nm-sized polystyrene beads and then covered with the cationic lipid DOTAP. The resulting particle size was measured to be around 207.3 ± 58.5 nm (Fig. S10, Supplementary Information). Then the prepared cancer diagnocode was treated to model breast cancer cells. Green signals originating from Alexa Fluor^®^ 488 traced the cellular uptake tendency in a time-dependent manner (Fig. S11a, Supplementary Information). Flow cytometry analysis demonstrated that cell types varied in the amount of diagnocodes they took up. Several studies have reported that there are significant variations in cellular behaviors according to the cell types^22^. Especially, nanoparticle uptake is quite different by the cell types^23^. Some factors are suggested to be responsible for this phenomenon, but the exact mechanism is still not clearly understood. One of the suggested contributing factors is the surface area of cells. Depending on the cell type, the cellular morphology would vary, leading to different surface areas. This difference may influence on the interaction between the cell and treated materials during their contact. Next, the way of internalization of nanoparticles is also quite cell specific. The cell specific feature of internalization can cause serious problems in diagnostic and therapeutic perspectives. The type of cells that are more likely to internalize external particles, might send off biased signals by taking internalization routes other than the intended strategic route designed for the treated diagnostic material. In contrast, those that do not tend to internalize external particles cannot give off enough signals because of absolute lack of internalized diagnostic materials.

Next, the ratio of Cy5^TM^ to Alexa Fluor^®^ 488 signal was measured. Interestingly, signal ratios remained constant, even in model cancer cells (Fig. S11b, Supplementary Information). These results were similar to solution-based *in vitro* findings where the signal was also independent of the length of diagnocode treatment. The steady signal indicated that, in most cancer cells, the diagnocode system worked as expected within the cytosolic environment. However, a slight increase in relative signals was seen after 4 hours of treatment in cancer types MCF-7 and SK-BR-3, suggesting the possibility of degradation of L-DNA structure by intracellular enzymes, resulting in unwanted signal release. Accordingly, the effectiveness of the diagnocodes for verifying cancer within a cytosolic environment was confirmed, while it should be analyzed within 2 hours of incubation to avoid additional damage to DNA molecules and to minimize unexpected errors.

### *In Situ* Cancer Diagnosis of Fluorescent Tile DNA Diagnocode System

**Figure 4:**
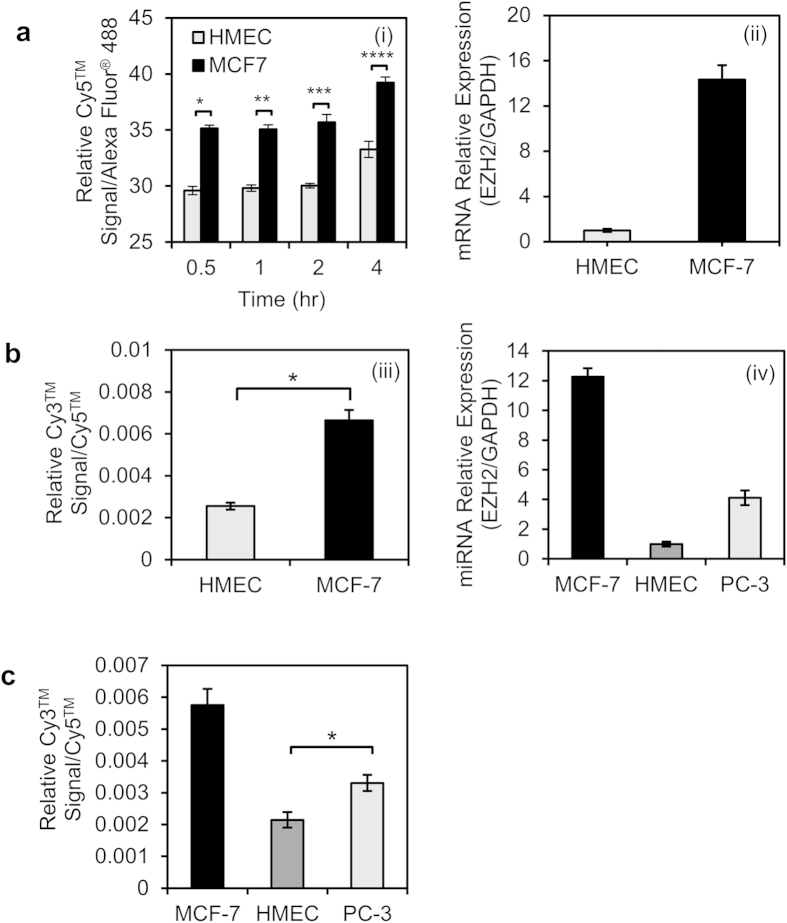
Cancer diagnosis using fluorescent diagnocode system using L-DNA and qRT-PCR for the detection of cytosolic RNA. (**a**) Flow cytometry was used to differentiate model cancer cells (MCF-7) from normal cells (HMEC), by targeting mRNA (EZH2) using diagnocode (i). Cy5^TM^ was used as the indicator while Alexa Fluor^®^ 488 was used as the standard. The cells were well distinguished regardless of diagnocode treatment time. *p* < 0.001 for * and *p < 0.01 for *** and ****. qRT-PCR (ii) confirmed that higher concentrations of EZH2 mRNA were being synthesized within the cytosol. (**b**) Cancer diagnosis by miRNA (miR21) detection using diagnocode. Cy3^TM^ was used as the indicator while Cy5^TM^ was the standard (iii). Cancer cells overexpressing miR21 (MCF-7) were clearly distinguished from control cells (HMEC) (*p < 0.01). qRT-PCR data confirmed the same pattern (iv). (**c**) Cancer differentiation using diagnocode. Three cell types were investigated, each modeling a different stage of cancer: malignant (MCF-7), benign (PC-3), and normal (HMEC) (*p < 0.05). The results corresponded with the qRT-PCR data shown in (iv).

Cancer diagnocodes were finally verified for rapid and *in situ* real-time diagnosis of two breast cell types (MCF-7 cancer cells overexpressing the EZH2 protein^24^ and normal human mammary epithelial cells (HMEC) as a negative control). MCF-7 cells exhibited higher Cy5^TM^ than Alexa Fluor^®^ 488 signals, and a greater overall signal strength than HMEC over the time periods examined (Fig. 4a), indicating that the prevalence of EZH2 mRNA in MCF-7 cells with sudden disruption of looped structures. Diagnocodes were able to distinguish cancer cells from normal ones within 30 minutes based on cytosolic RNA expression, as confirmed by qRT-PCR (Fig. 4a,ii), since the cancer cells produced more RNA molecules than normal cells. The relative intensity of diagnocodes were measured with flow cytometry. Especially, Cy3 is commonly excited with 488 nm wavelength of laser source in flow cytometry, even the optimal exitation wavelength is 520 nm. Thus, absolute value of relative intensity of Cy3/Cy5 was lower than the Cy5/AF488 pair.

The cancer diagnocodes were further evaluated using the cancer-specific microRNA (miRNA) miR21, which is overexpressed in most types of cancer cells (e.g., MCF-7)^25^. After 30 minutes of treatment, the relative signal ratios of Cy3^TM^ to Cy5^TM^ on diagnocodes in MCF-7 and HMEC cells were monitored and the results were compared using qRT-PCR (Fig. 4b, iii and iv). In contrast to the mRNA-detecting diagnocode, which used Cy5^TM^ as an indicator and Alexa Fluor^®^ 488 as a standard, the miR21 diagnocode contained Cy3^TM^ and Cy5^TM^ as the indicator and standard fluorophores, respectively. These diagnocodes were able to detect cytosolic miRNA, resulting in successful differentiation of the cell types, as confirmed by qRT-PCR. Diagnocodes also distinguished between MCF-7 and SK-BR-3 cells, which are derived from different cancer origins. They were again substantiated by qRT-PCR (Fig. S12a and S12b, Supplementary Information). Diagnocodes detected miRNA in multiple cancer cell types simultaneously (Fig. 4c; confirmed by qRT-PCR in Fig. 4b,iv). Interestingly, the relative signals exhibited by PC-3 cancer diagnocodes were moderate compared to those of the other model cancer cells and were much closer to signals observed in HMEC cells, indicating that PC-3 was a less malignant cancer than MCF-7 with regard to miR21 expression. These findings demonstrate the effectiveness of the diagnocodes at diagnosing a broad range of cell types.

## Discussion

In this study, we have created a new rapid and real-time cancer diagnostic code based on structural DNA nanotechnology and FRET phenomena that offers higher sensitivity and selectivity. By virtue of the heterogeneity of the DNA structure, two or three different fluorescent color dyes and a quencher were complexed, working together via FRET in response to oligohybridization of cytosolic RNA markers in cytosolic environment of the target breast cancer cells. Signal strength was mainly dependent on complementary RNA mass and was unrelated to diagnocode concentration or treatment time. Thus, any post-treatment optimization that varied by cell type was unnecessary. This novel approach offers a significant advancement for preventive material medicine over conventional tool kits by providing unbiased outcomes and enhanced diagnostic accuracy irrespective of the type of target cell and careful considerations about both time and amount of cell treatment. Therefore, the diagnocode system can easily be applied to a wide range of cell types, simplifying the entire diagnostic process. The target breast cancer model, MCF-7, was distinguished from normal breast cells, HMEC, other cancer types, SK-BR-3, within 30 minutes based on relative color intensity ratios. The ability to discriminate the degree of cell malignancy offers the possibility of rapid cancer diagnosis and monitoring in a clinical setting. The diagnocode cancer diagnostic system shows potential for multiplexed and personalized cancer diagnosis in the near future.

## Materials and Methods

### Y-DNA and L-DNA Preparation

Lyophilized oligonucleotides (Integrated DNA Technologies, Inc., Coralville, Iowa, USA) composed of Y and L-DNA blocks were separately dissolved in TE buffer (10 mM Tris, pH 7.5, 0.1 mM EDTA). The optical density (OD) of each solution was measured using a UV-Vis photometer (BioPhotometer Plus, Eppendorf), and the concentrations (in μg/μl) of oligonucleotides were calculated as described previously^16^. The oligonucleotide solutions were mixed at equal molar ratios to a final concentration of 6 μM. The mixture was put into a thermocycler (Mastercycler^®^ Pro, Eppendorf) and then exposed to stepwise temperature variation to anneal the oligonucleotides: samples were heated to 95 °C for two minutes, cooled to 65 °C for five minutes and further cooled to 60 °C for five minutes. The temperature was then reduced by 1 °C per minute until it reached 20 °C. The samples were stored at 4 °C until use.

### Preparation of Cancer Diagnocodes

Streptavidin coated PS bead stock solution (Bangs Laboratories, Inc., Fishers, IN, USA) was diluted in PBS (0.05% Tween 20) at a 1% volumetric ratio. The solution containing biotin labeled tile DNA (6 μM) was added to the diluted beads (10:1 volumetric ratio) and the mixture was incubated for an hour at room temperature, which allowed the tile DNA to be immobilized at the surface of PS beads through streptavidin-biotin chemistry. The liposome solution (2.5 mg/ml) was then added to the mixture at a 1:3 volumetric ratio and the solution was incubated for an additional hour at room temperature. After the reaction, the sample was centrifuged (10,000 g, 30 minutes, 4 °C), washed once with PBS and resuspended in 100 μl of PBS.

### Statistical Analysis

Results in different cancer cell types were compared using two-tailed student’s t-tests, and *p* ≤ 0.05 was considered significant (n ≥ 3). To compare multiple pairs of signal ratios (e.g., either Cy5^TM^:Alexa Fluor^TM^ 488 or Cy3^TM^:Cy5^TM^) at different time periods in the same sample, ANOVA test was used to determine whether the pairs were significantly different from each other. Pairs were considered to have no significant difference at p > 0.05.

## Additional Information

**How to cite this article**: Park, K. S. *et al.* A Fluorescent Tile DNA Diagnocode System for *In Situ* Rapid and Selective Diagnosis of Cytosolic RNA Cancer Markers. *Sci. Rep.* **5**, 18497; doi: 10.1038/srep18497 (2015).

## Supplementary Material

Supplementary Data 1

Supplementary Data 2

Supplementary Information
