# Supplementary material for: A Fluorescent Tile DNA Diagnocode System for In Situ Rapid and Selective Diagnosis of Cytosolic RNA Cancer Markers
Source: Sci Rep. 2015 Dec 18;5:18497. doi: 10.1038/srep18497 (PMC4683441; doi:10.1038/srep18497)
Supplement: Supplementary Data 1 [file srep18497-s1.doc]

#include <stdio.h>

#include <math.h>

int main() {

int num, a, b, c, rea, reb, rec, k, i, ca, cb, cc;

double gae;

printf("what is generation number?");

scanf("%d", &num);

printf("generation number : %d\n",num);

gae = pow(2.0,num);

a = (int) gae;

printf("dye number : %lf", gae);

i = 0;

printf("\n");

printf("lists");

printf("\n");

while(a > (int)gae/3) {

b = (int) gae -a;

c = 0;

while(b >= c){

if(b > a){

goto Fin;

}

k =2;

while(b>=k){

rea = a%k;

reb = b%k;

rec = c%k;

if(rea==0){

if(reb==0){

if(rec==0){

goto Fin;

}

}

}

k = k+1;

}

if(b==0){

goto Fin;

}

if(a>b){

if(b>c){

ca = a;

cb = b;

cc = c;

while(ca>0){

printf("R");

ca--;

}

while(cb>0){

printf("G");

cb--;

}

while(cc>0){

printf("B");

cc--;

}

printf(" ");

i = i+1;

ca = a;

cb = b;

cc = c;

while(ca>0){

printf("R");

ca--;

}

while(cb>0){

printf("B");

cb--;

}

while(cc>0){

printf("G");

cc--;

}

printf(" ");

i = i+1;

ca = a;

cb = b;

cc = c;

while(ca>0){

printf("B");

ca--;

}

while(cb>0){

printf("R");

cb--;

}

while(cc>0){

printf("G");

cc--;

}

printf(" ");

i = i+1;

printf("\n");

ca = a;

cb = b;

cc = c;

while(ca>0){

printf("B");

ca--;

}

while(cb>0){

printf("G");

cb--;

}

while(cc>0){

printf("R");

cc--;

}

printf(" ");

i = i+1;

ca = a;

cb = b;

cc = c;

while(ca>0){

printf("G");

ca--;

}

while(cb>0){

printf("B");

cb--;

}

while(cc>0){

printf("R");

cc--;

}

printf(" ");

i = i+1;

ca = a;

cb = b;

cc = c;

while(ca>0){

printf("G");

ca--;

}

while(cb>0){

printf("R");

cb--;

}

while(cc>0){

printf("B");

cc--;

}

printf(" ");

i = i+1;

printf("\n");

}

else{

ca = a;

cb = b;

cc = c;

while(ca>0){

printf("R");

ca--;

}

while(cb>0){

printf("B");

cb--;

}

while(cc>0){

printf("G");

cc--;

}

printf(" ");

i = i+1;

ca = a;

cb = b;

cc = c;

while(ca>0){

printf("G");

ca--;

}

while(cb>0){

printf("R");

cb--;

}

while(cc>0){

printf("B");

cc--;

}

printf(" ");

i = i+1;

ca = a;

cb = b;

cc = c;

while(ca>0){

printf("B");

ca--;

}

while(cb>0){

printf("G");

cb--;

}

while(cc>0){

printf("R");

cc--;

}

printf(" ");

i = i+1;

printf("\n");

}

}

else{

if(b>c){

ca = a;

cb = b;

cc = c;

while(ca>0){

printf("G");

ca--;

}

while(cb>0){

printf("R");

cb--;

}

while(cc>0){

printf("B");

cc--;

}

printf(" ");

i = i+1;

ca = a;

cb = b;

cc = c;

while(ca>0){

printf("R");

ca--;

}

while(cb>0){

printf("B");

cb--;

}

while(cc>0){

printf("G");

cc--;

}

printf(" ");

i = i+1;

ca = a;

cb = b;

cc = c;

while(ca>0){

printf("B");

ca--;

}

while(cb>0){

printf("G");

cb--;

}

while(cc>0){

printf("R");

cc--;

}

printf(" ");

i = i+1;

printf("\n");

}

else {

printf(" ");

printf("\n");

}

}

Fin:;

b= b-1;

c = (int) gae-a-b;

}

a = a-1;

}

printf("\n");

printf("the number of kinds of barcode : %d", i);

scanf("%d",&i);

return 0;

}
