# Supplementary material for: A Fluorescent Tile DNA Diagnocode System for In Situ Rapid and Selective Diagnosis of Cytosolic RNA Cancer Markers
Source: Sci Rep. 2015 Dec 18;5:18497. doi: 10.1038/srep18497 (PMC4683441; doi:10.1038/srep18497)
Supplement: Supplementary Data 2 [file srep18497-s2.doc]

% filename = 'RB.png'; nx = 30; ny = 15;

cd('KSP_DNA nanobarcode')

%%% B-3 G-2 R-1

Ms = meshgrid(1:100,1:100);

prompt = 'Red - 1; Green - 2; Blue - 3; Please input in this format e.g. [1 2 3] with spaces';

result = input(prompt);

prompt2 = 'Intensity e.g. 0.5 (should be between 0 and 1)';

result2 = input(prompt2);

prompt3 = 'Individual Intensity e.g. [ 0.5 1 0.5] (should be between 0 and 1)';

result3 = input(prompt3);

if ~isempty(result)

nR = length(find(result == 1));

nG = length(find(result == 2));

nB = length(find(result == 3));

nrgb = [nR nG nB].*result3;

nrgb = nrgb./max(nrgb)*result2;

nrgb = round(nrgb*2^8);

Ms(:,:,1) = nrgb(1);

Ms(:,:,2) = nrgb(2);

Ms(:,:,3) = nrgb(3);

X = uint8(Ms);

figure(5); clf; imshow(X);

prompt = 'Would you like to save this image? [Yes (9) No (0)]';

ynres = input(prompt);

if ynres == 9

imwrite(X, ['Im' int2str(result) '.bmp'])

end

end

return;
