# Supplementary material for: A Fluorescent Tile DNA Diagnocode System for In Situ Rapid and Selective Diagnosis of Cytosolic RNA Cancer Markers
Source: Sci Rep. 2015 Dec 18;5:18497. doi: 10.1038/srep18497 (PMC4683441; doi:10.1038/srep18497)
Supplement: Supplementary Information [file srep18497-s3.doc]

**Supplementary Information for**

**A Fluorescent Tile DNA Diagnocode System for *In Situ* Rapid and Selective Diagnosis of Cytosolic RNA Cancer Markers**

*Kyung Soo Park, Seung Won Shin, Min Su Jang, Woojung Shin, Kisuk Yang, Junhong Min, Seung-Woo Cho, Byung-Keun Oh, Jong Wook Bae, Sunghwan Jung, Jeong-Woo Choi*, and Soong Ho Um**

***E-mail:** [**jwchoi@sogang.ac.kr**](mailto:jwchoi@sogang.ac.kr)**;** [**sh.um@skku.edu**](mailto:sh.um@skku.edu)**.**

K. S. P. And S. W. S. contributed equally to this work.

**CONTENTS**

**Suppplementary Figures and Table**

Suppplementary Figure 1 Temperature variation set-up for tile DNA synthesis

Suppplementary Figure 2 Gel-electrophoretic migration shift assay of tile DNA

Suppplementary Figure 3 Signal analysis of amplified fluorescence color codes using both confocal microscopy and flow cytometry.

Suppplementary Figure 4 Calculation for denoting codes

Suppplementary Figure 5 Flow cytometry histogram of the fluorescence intensity related to the number of fluorescent code labeled T-DNA molecules on microbeads

Suppplementary Figure 6 Flow cytometry and confocal microscopic analysis of physically mixed Y-DNA-tagged beads and chemically-conjugated Y-DNA-tagged beads

Suppplementary Figure 7 Increment of indicator fluorescence (Cy5^TM^) from L-DNA after the addition of 10 and 100 molar folds of complementary oligonucleotide compared to the L-DNA

Suppplementary Figure 8 Temperature conditions for the prevention of L-DNA deformation in diagnocodes

Suppplementary Figure 9 Evaluation of characteristics of liposomes, including size, surface charge and morphological properties

Suppplementary Figure 10 Hydrodynamic diameter of DOTAP-enveloped diagnocodes

Suppplementary Figure 11 Analysis of cellular uptake of diagnocodes over time

Suppplementary Figure 12 Comparison of miR21 expression in two different breast cancer cell lines: SK-BR-3 and MCF-7

Suppplementary Table 1 Sequence information for all oligonucleotides for the construction of title DNA nanostructures

**Suppplementary Notes**

Studies on physiochemical characteristics of the Y-DNA (in the context, it contains computer codes regarding MATLAB and C++ but they are enclosed separately as a suppplementary data in the attached file box)

**Suppplementary Methods**

1. T-DNA preparation
2. Agarose gel electrophoresis
3. Preparation of unilamellar lipid bilayers
4. Measuring size and zeta potential of particles using dynamic light scattering (DLS)
5. Flow cytometry analysis
6. Transmission electron microscopic (TEM) analysis of cellular uptake of cancer diagnocodes
7. Quantitative Real-Time Polymerase Chain Reaction (qRT-PCR)

**Suppplementary References**

**Suppplementary Figures and Table**

**
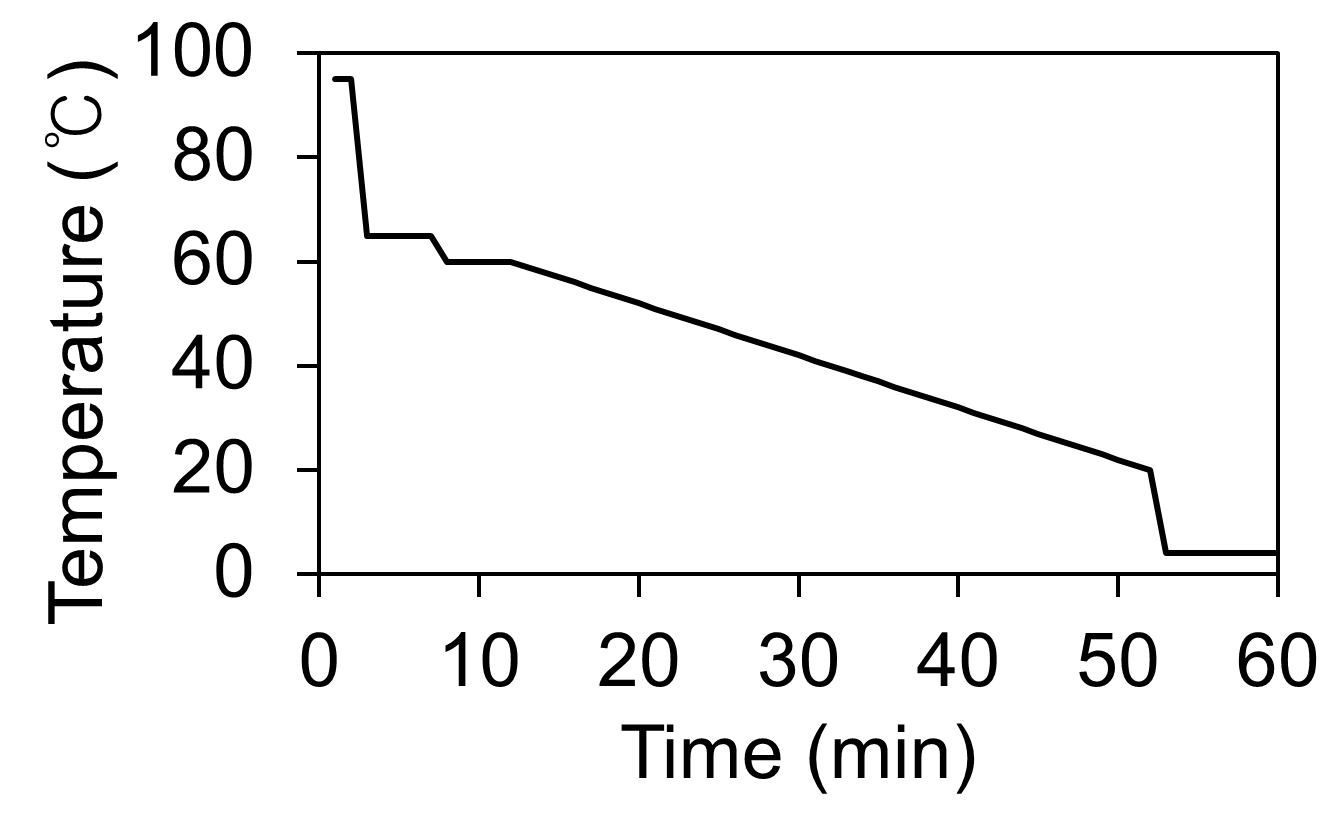
**

**Figure S1.** Temperature variation set-up for tile DNA synthesis. The samples were heated at 95 ^o^C for two minutes, cooled to 65 ^o^C for five minutes and further cooled to 60 ^o^C for five minutes. The samples were then cooled by 1 ^o^C per minute until the temperature reached 20 ^o^C, and then further cooled to 4 ^o^C for storage.

**
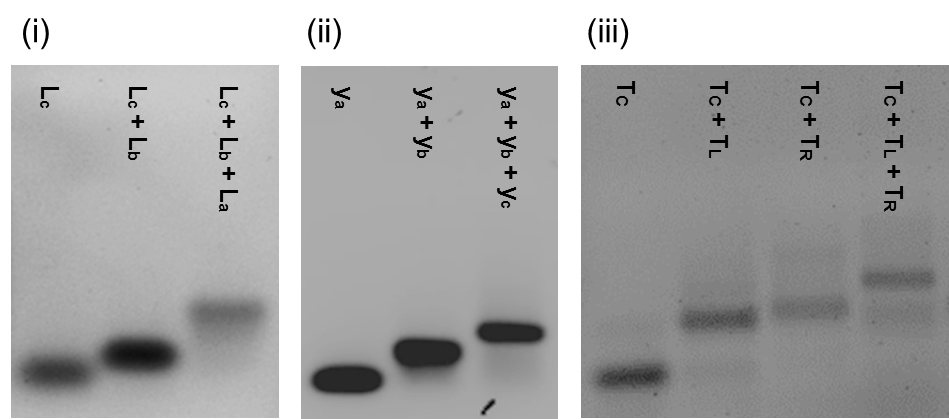
**

**Figure S2.** Gel-electrophoretic migration shift assay of tile DNA nanostructures: L-DNA (i), Y-DNA (ii) and T-DNA (iii). Stepwise movements of each band indicate sequential formation of the synthesized DNA structures. Oligonucleotide sequences are compiled in Table S1.


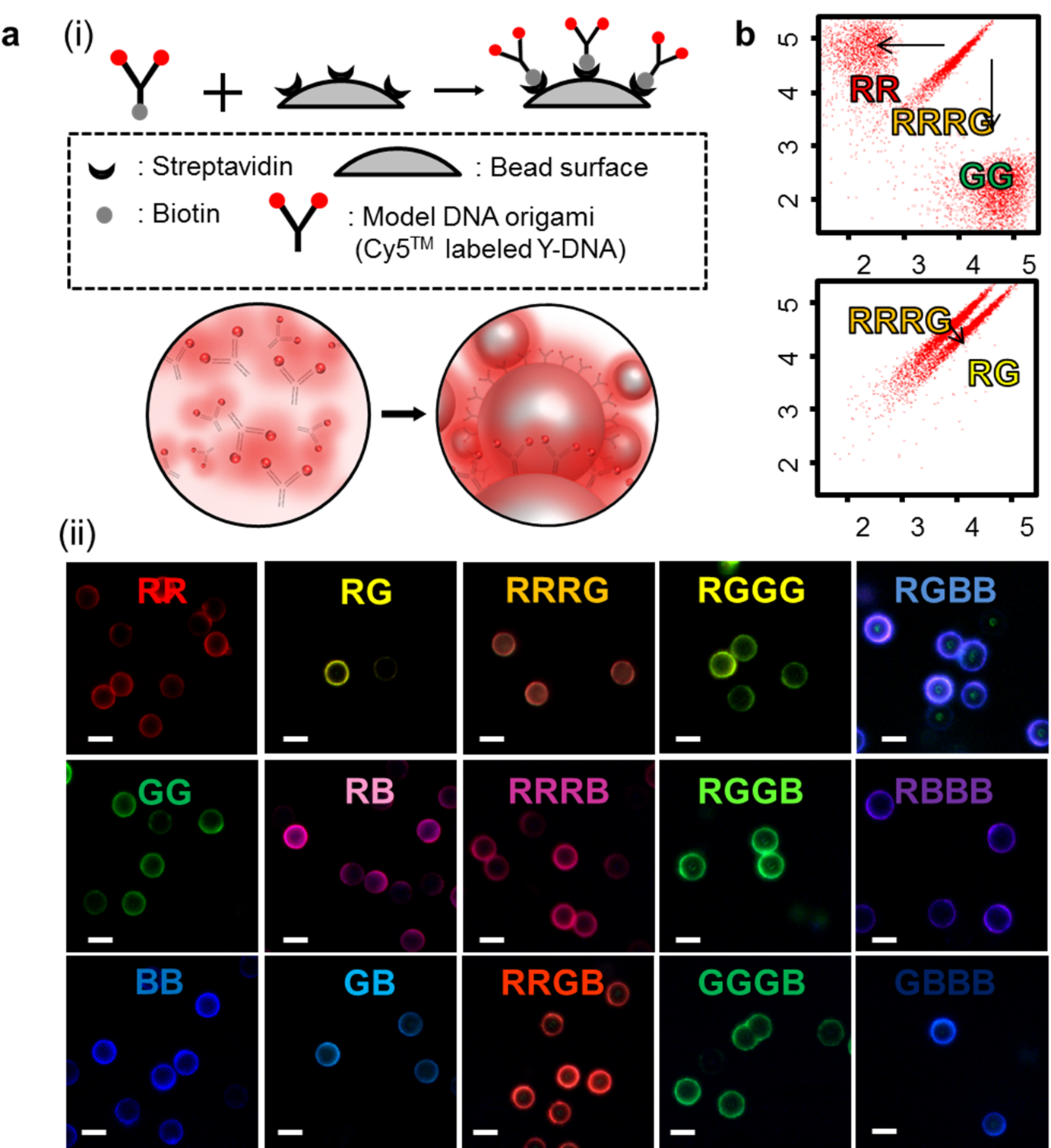


**Figure S3.** Signal analysis of amplified fluorescence color codes using both confocal microscopy and flow cytometry. (**a**) Schematic drawings showing the synthetic process of signal amplification (*i*). Color encoded tile DNA nanostructures were conjugated to 5.2-µm silica microbeads for signal amplification, as observed with a confocal microscope. Three different fluorescent dyes were incorporated into either a Y-DNA or a second-generation T-DNA: Cy5^TM^ for red, 6-FAM^TM^ for green and TEX^TM^ 615 for blue. The confocal images were brought to the bottom side and tagged as (*ii*). The scale bars in (*ii*) correspond to 5 μm. (**b**) Flow cytometry analysis of encoded tile DNA was dependent on the number ratio of the fluorescent dyes used. In two-dimensional plots, the ratio shifts either to the x or y axis. The value in each axis is expressed in log scale. Arrows indicate the direction of the color shifts after code replacements.

**
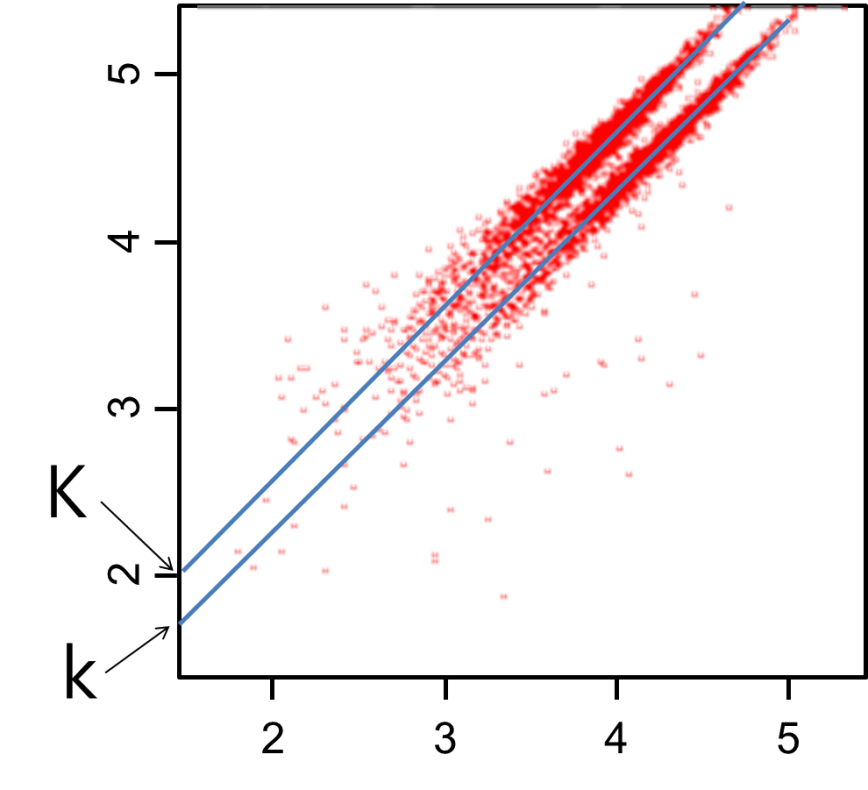
**

**Figure S4.** Calculation for denoting codes. Fluorescence signals from 6-FAM^TM^ were plotted along the x-axis while Cy5^TM^ signals were plotted along the y-axis. Both axes are expressed in log scale. The codes were calculated using the y-intercept on the flow cytometry graph. The relative molar ratio of Red (R) to Green (G) code was designated “m”. K/m was calculated based on the y-intercepts of the histogram, resulting in k. The code (1GmˊR) was decoded by a relative comparison with an arbitrary standard code (1GmR, m=3). Note that m and mˊ were the relative number of R for each G. K and k were defined as y-intercepts of the graphs of the standard code and the code of interest, respectively.

The following equation was derived:

m:mˊ = 10^K^:10^k^

According to the graph, K=2 and k=1.5. Thus, m:mˊ≒100:32, where m=3. Therefore, mˊ≒1.


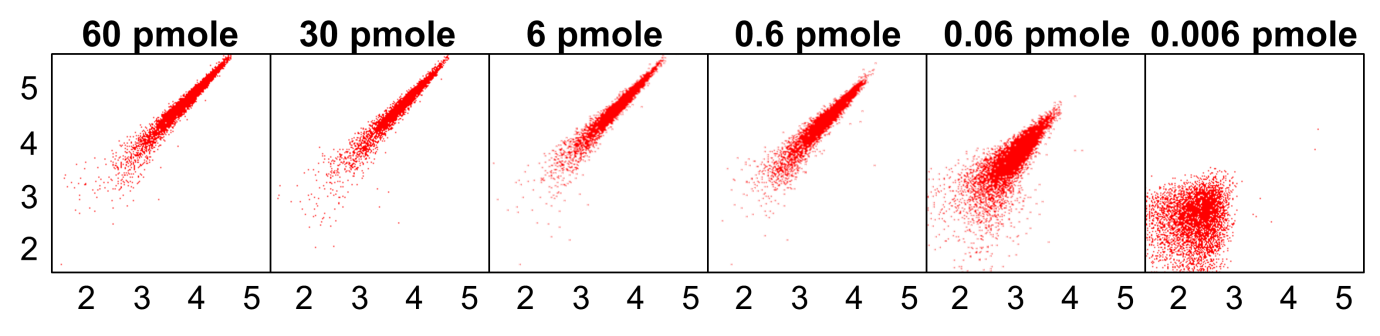


**Figure S5.** Flow cytometry histogram of fluorescence intensity related to the number of fluorescent code-labeled T-DNA molecules on microbeads. Signal intensity varied depending on the concentration of tile DNA in the microbead reaction mixture, corresponding to the signal shift to the right in the histogram. Fluorescence signals from 6-FAM^TM^ are plotted on the x-axis while those of Cy5^TM^ are plotted on the y-axis. Values are expressed in moles of fluorescent dye-labeled (Cy5^TM^ and 6-FAM^TM^ in 3:1 ratio) T-DNA that reacted with 0.1156 pmole of 5.2 μm silica beads. Both axes are expressed in log scale.


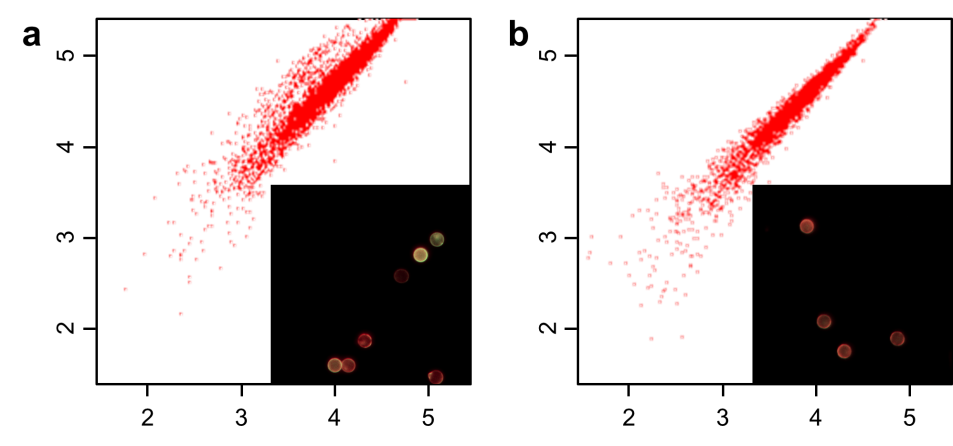


**Figure S6.** Flow cytometry and confocal microscopic analysis (insets) of physically mixed Y-DNA-tagged beads (**a**) and chemically-conjugated Y-DNA-tagged beads (**b**). The x-axis corresponds to fluorescence signals from 6-FAM^TM^ while the y-axis corresponds to Cy5^TM^ signals. Both axes are expressed in log scale.

**
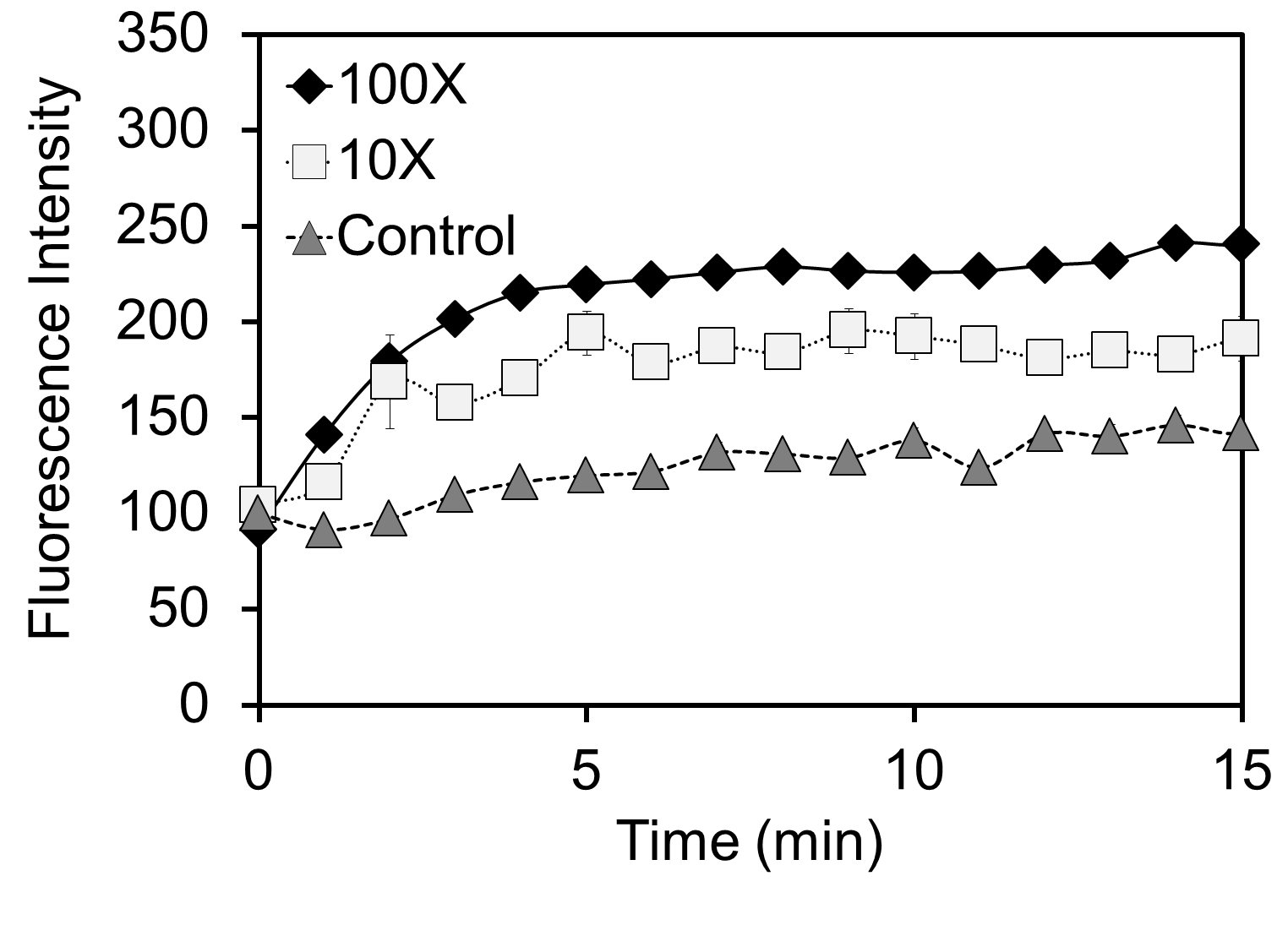
**

**Figure S7.** Increment of indicator fluorescence (Cy5^TM^) from L-DNA after the addition of 10 and 100 molar folds of complementary oligonucleotide compared to L-DNA. The signal intensity was proportional to the amount of the complementary oligonucleotide added. The fluorescence increase was observed as soon as the complementary strand was added, and reached saturation in less than five minutes. The reaction was conducted in 37 ^o^C.


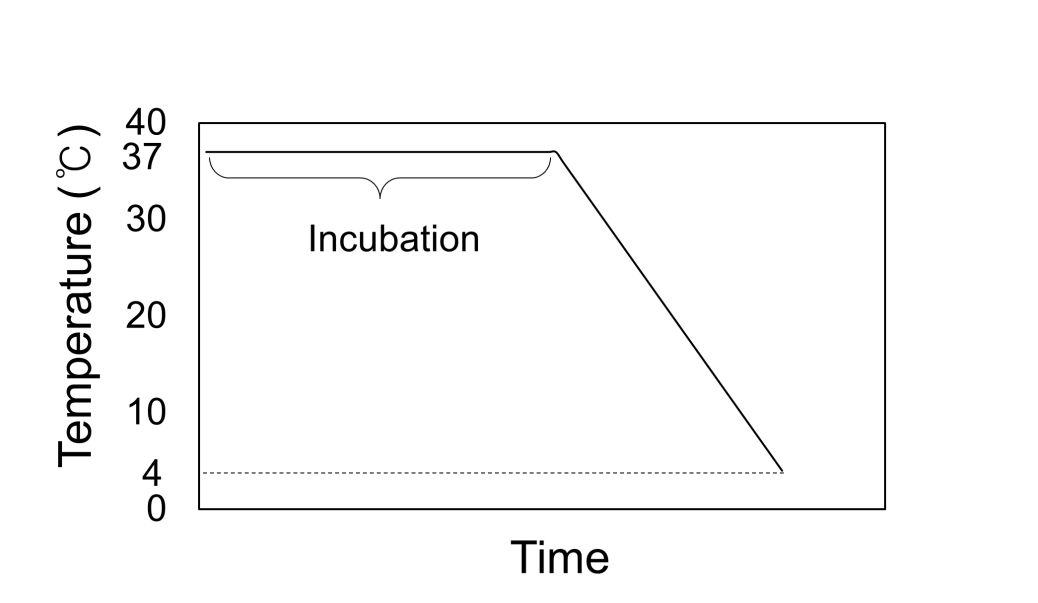


**Figure S8.** Temperature conditions for the prevention of L-DNA deformation in diagnocodes. After treatment with diagnocodes, cuvettes were incubated for varying lengths of time at 37 ^o^C. The temperature was then slowly reduced to 4 ^o^C by decreasing 1 ^o^C every 30 seconds.

**
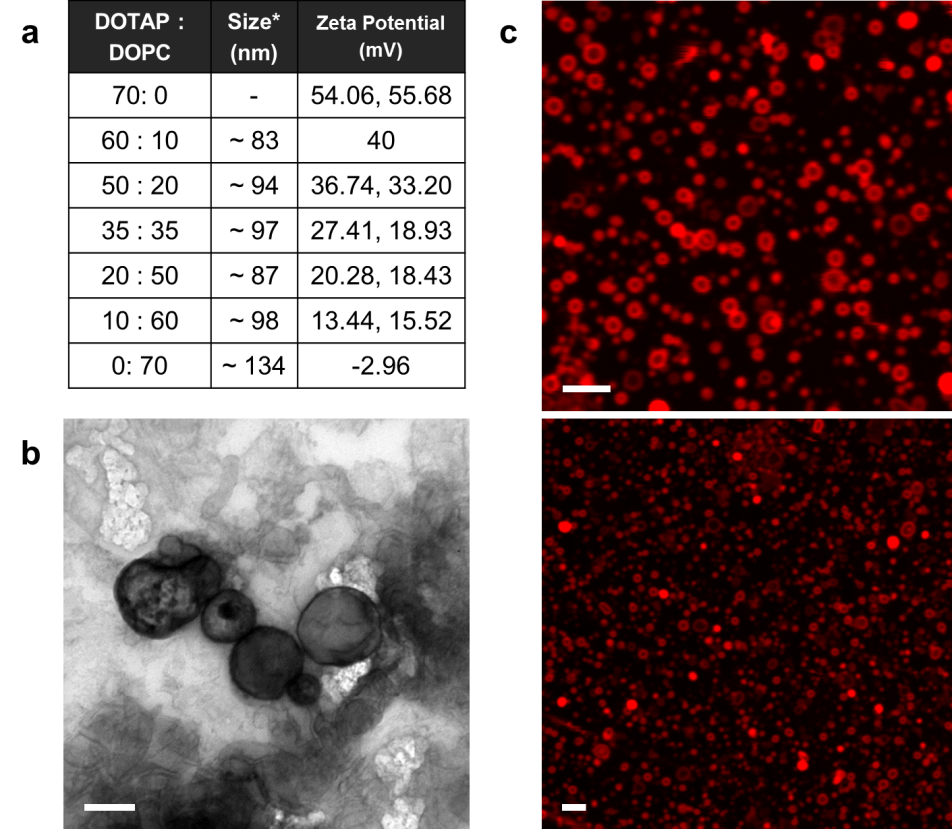
**

**Figure S9.** Evaluation of characteristics of liposomes, including size, surface charge and morphological properties. The variable ratios between DOTAP and DOPC were measured by weight%, while the remaining 30% of each lipid bilayer was cholesterol. (**a**) Size and surface charge of liposomes with various composition ratios, measured using ELS. While the size distribution remained constant at approximately 100 nm regardless of the composition ratio, the surface charge became more positive as the percentage of DOTAP increased. (**b**) Transmission electron microscopic images stained with uranyl acetate showed that the liposomes were approximately 100 nm as measured by ELS and appeared in a single layer. According to the technical instructions of Avanti Polar Lipids, lipid extrusion with a 100 nm-pore sized filter may produce 120-140 nm unilamellar vesicles (LUV). (**c**) In the confocal microscopic image, Texas Red® DHPE was selectively incorporated into the total lipid composition. The scale bar is labeled in each figure. Scale bars are 100 nm for b and 2 µm for c.

**
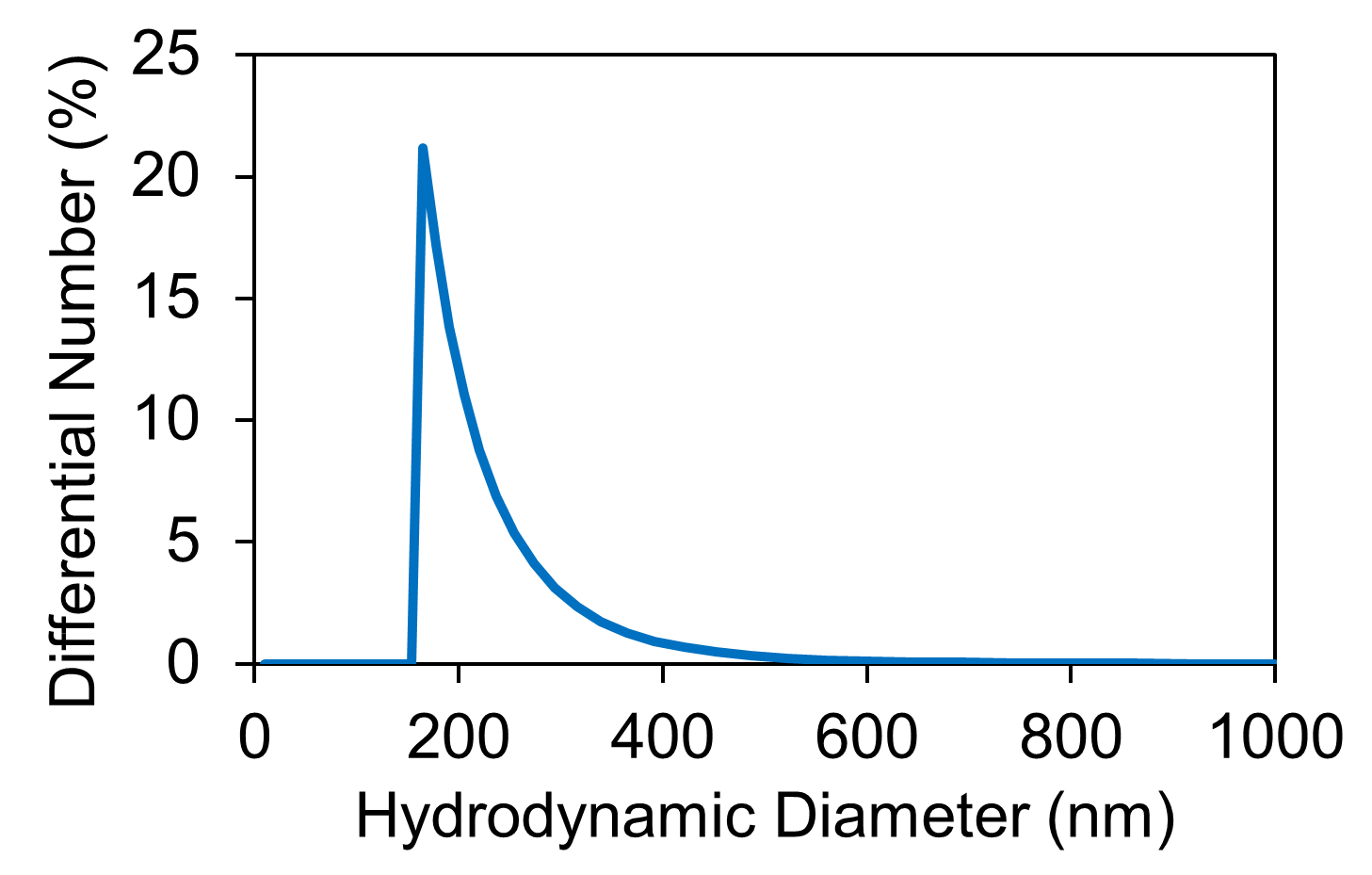
**

**Figure S10.** Hydrodynamic diameter of DOTAP-enveloped diagnocodes. Size was measured using dynamic light scattering (DLS). The mean hydrodynamic diameter of the particles was 207.3 ± 58.5 nm. Each size measurement was conducted in triplicate, and each measurement was an accumulation of 50 analyses using a 653-nm laser source. The data was converted into a nano-meter scale using the accumulation method provided by ELS-Z software (Otsuka Electronics Co.)

**
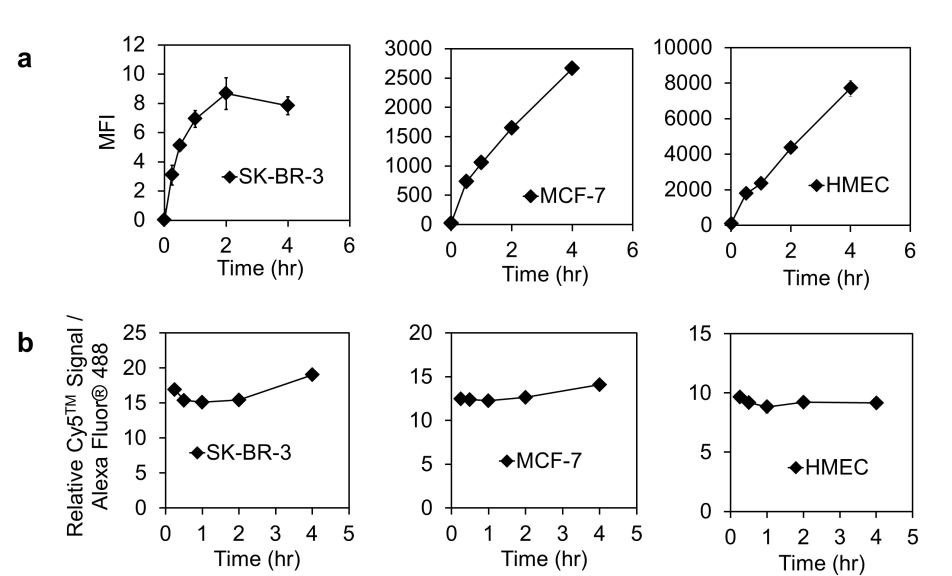
**

**Figure S11.** Analysis of cellular uptake of diagnocodes over time. (**a**) The model cells exhibited a time-dependent increase in cellular uptake. Cellular uptake of diagnocodes were assessed with mean fluorescence intensity (MFI). (**b**) Although the number of particles increased during incubation, the Cy5^TM^ to Alexa Fluor® 488 signal ratio remained steady, not showing significant difference between pairs of ratios at different time periods. However, a slight increase (significant difference) in relative signals was seen after 4 hours of treatment in the cases of cancer types MCF-7 and SK-BR-3, suggesting the possibility of degradation of the L-DNA by intracellular enzymes that resulted in unwanted signal release. All flow cytometry analysis was conducted independently.

**
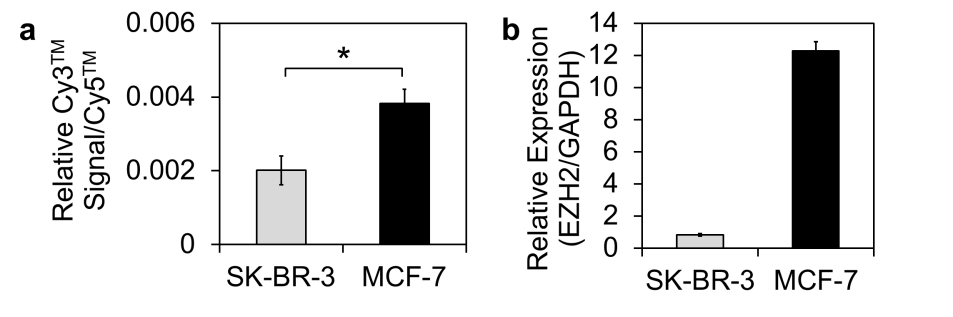
**

**Figure S12.** Comparison of miR21 expression in two different breast cancer cell lines: SK-BR-3 and MCF-7. Flow cytometry (a) and qRT-PCR (b) confirmed that the two cancer cells were indeed significantly different cell types (t-test,* *p* < 0.05).

**Table S1.** Sequence information for all oligonucleotides for the construction of tile DNA.

| **Strand name** | | **Chemical modification at a 5 primed end** | **Body sequence** |
| --- | --- | --- | --- |
| y_a_ | 1 | Biotin | AGGCTGATTCGGTTCATGCGGATCCA-3’ |
|  | 2 | Phosphorylation/AGTC/ |  |
|  | 3 | Phosphorylation/GCAT/ |  |
|  | 4 | Phosphorylation/ATGC/ |  |
| L_a_ | 1 | Iowa Black® RQ/GCGAGGCCAGACTGGGAAGAAATCTGCTCGC/Cy5^TM^/ |  |
|  | 2 | Iowa Black® RQ/GCGAGTCAACATCAGTCTGATAAGCTACTCGC/Cy3^TM^/ |  |
| L_b_ | 1 | Alexa Fluor® 488 | CTTACGGCGAATGACCGAATCAGCCT-3’ |
| y_b_ | 1 | 6-FAM^TM^ |  |
|  | 2 | Cy5^TM^ |  |
|  | 3 | TEX^TM^ 615 |  |
|  | 4 | Phosphorylation/GACT/ |  |
| L_c_ | 1 | Biotin | TGGATCCGCATGACATTCGCCGTAAG-3’ |
| y_c_ | 1 | Cy5^TM^ |  |
|  | 2 | 6-FAM^TM^ |  |
|  | 3 | TEX^TM^ 615 |  |

**Suppplementary Notes**

*Studies on physiochemical characteristics of the Y-DNA*: In order to study the physicochemical characteristics of the L-DNA, three branched DNA forms were prepared: Y-DNA, T-DNA and L-DNA. Briefly, by adding cohesive ends to the hands of the Y-DNAs, these DNA nanostructures are linked, after which it becomes more intense through ligation using T4 ligase. The DNA structures contained fluorescence tags Cy5^TM^ for red (R), 6-FAM^TM^ and Alexa Fluor® 488 for green (G), and TEX^TM^ 615 and Cy3^TM^ for blue (B). Tile DNA structures were synthesized by complementary hybridization of each oligonucleotide precursor by placing the mixtures at varying temperatures based on previous studies (Figure S1, Suppplementary Information).^[1]^ The sequence of each oligonucleotide is shown in Table S1 found in Suppplementary Information section. The formation of tile DNA was confirmed through gel-electrophoresis (Figure S2, Suppplementary Information).

To evaluate the effectiveness of the anisotropic characteristic of the Y-shaped DNA structure, fluorescent dyes were first attached to two of the three hands of the Y-DNA, and a biotin was attached to the other hand. While maintaining the biotin conjugated portion, it resulted in 6 color combinations when three different dyes were adopted. Second-generation T-DNA was used to diversify the combination of fluorescent dyes attached to the DNA nanostructure which added nine more to the number of color combination. It is expected that the use of higher-generation T-DNA will allow an even larger number of colors to be combined.

The resulting Y- or T-DNA were conjugated to 5.2-μm streptavidin-coated silica beads for confocal microscopic observation (Figure S3a). According to the dyes used and their ratio, the fluorescence exhibited from the beads well demonstrated distinct colors, indicating that the DNA structures defined the directions to which the dyes are attached as programed. The expected colors and the number of the combinations could be predicted in advance using MATLAB (Matrix Laboratory) and C++ language software, respectively (see Suppplementary Data for more information on both programs).

Subsequently, the color expression by the DNA nanostructures was further investigated by flow cytometry. The use of two different fluorescent dyes (in this case, Cy5^TM^ and 6-FAM^TM^) produced a sharp linear graph. Depending on the emission intensities of the selected fluorescent dyes and their ratios, shifts occurred along either the x- or y-axis (Figure S3b). A simple calculation was used to denote each color ratio (Figure S4, Suppplementary Information). Varying the molar ratios of the three fluorescent dyes on T-DNAs produced distinct colors, and signal intensities could be precisely controlled by adjusting the concentration of T-DNA during microbead conjugation (Figure S5, Suppplementary Information). The effectiveness of the biochemically realized color combination was confirmed by comparison with a sample realized by physically mixing samples of different colors; one green and three reds, 1G3R. In both cases, the DNA nanostructures were conjugated to 5.2-μm-diameter silica beads, followed by flow cytometry and confocal microscopic examination. For the physically mixed sample, two different Y-DNAs, a 1G1R and a 2R color combination, were simultaneously mixed to a solution during the conjugation. The mixture of two color-labeled Y-DNAs resulted in relatively broader and uneven color distribution as compared to fluorescent dye-tagged T-DNA (Figure S6, Suppplementary Information), illustrating the effectiveness of the DNA structures as frameworks for the expression of fluorescent color combinations.

**Suppplementary Methods**

1. *T-DNA preparation*: In order to create T-DNA, three Y-DNA blocks were ligated using T4 ligase (Promega, Madison, WI, USA). One Y-DNA block (core Y-DNA) had biotin attached to one of its hands and specific “sticky end” sequences attached to the other two hands to serve as bridges linking the other two Y-DNA blocks (signal Y-DNA). The two signal Y-DNAs had sticky ends that ligated to the core Y-DNA’s corresponding sticky ends on one arm and fluorophores attached to the other arm. The three Y-DNA blocks were combined at an equal molar ratio to a final concentration of 1.2 μM and mixed with T4 ligase in ligase buffer. Under the direction of the T4 ligase, the three Y-DNAs formed T-DNA in a preprogrammed manner. The resulting T-DNA were used without further purifications.
2. *Agarose gel electrophoresis*: Gel electrophoretic assays used 3% (wt/vol) agarose gels and were run in Tris-acetate-EDTA (TAE) buffer at pH 8.3-8.5. Approximately 0.8-1.0 µg of DNA was added to each well and the samples were run at 60 V for 40 minutes. Gels were stained with ethidium bromide (EtBr) solution for 10 minutes and fluorescence was analyzed using a gel-doc system.
3. *Preparation of unilamellar lipid bilayers*: All lipids were purchased from Avanti® Polar Lipids, Inc. 1,2-dioleoyl-sn-glycero-3-phosphocholine (DOPC), 1,2-dioleoyl-3-trimethylammonium-propane (DOTAP) and cholesterol (all dissolved in chloroform) were combined in a glass vial at various mass ratios (DOPC:DOTAP:Cholesterol = X:Y:30 wt%, X+Y = 70) for a total mass of 2.5 mg. The lipids were dried overnight in a room temperature desiccator and then rehydrated by adding 1 ml of phosphate buffered saline (PBS) for an hour with shaking by hand for 30 seconds every 10 minutes. The resulting multilamellar vesicles (MLV) were then extruded (Avanti® Polar Lipids, Inc.), and the solution was passed through a 100-nm polycarbonate filter at least 20 times. The resulting unilamellar liposome solution (2.5 mg/ml) was kept at 4 ^o^C for up to three days before use.
4. *Measuring size and zeta potential of particles using dynamic light scattering (DLS)*: Particles (6.96 x 10^11^ polystyrene particles and diagnocodes) were dispersed in 700 μl of PBS for size measurement and 4 ml of PBS for zeta potential measurement. The samples were analyzed using a dynamic light scattering instrument with a 653-nm laser source (ELS-Z, Otsuka Electronics, Co.). For liposome measurements, 20 μl of 25 mg/ml liposome solution (in PBS) were dispersed in an equal volume of PBS. The size measurement was conducted in triplicate and each measurement was an accumulation of 50 analyses. The accumulated data was converted and recalculated using an accumulation method on a nano-meter scale using ELS-Z software (Otsuka Electronics Co.). Zeta potential measurements were conducted in duplicate using the same instrument.
5. *Flow cytometry analysis*: Fifty thousand cells were seeded into each well of 24-well plate and incubated overnight at 37 ^o^C. Thirty microliters of the prepared diagnostic material solution was added to each well for 30 min, 1 hr, 2 hrs, or 4 hrs. The cells were collected in a trypsin/EDTA solution, washed twice with PBS and suspended in 100 μl of 10% formaldehyde in PBS (vol%). The cells were then placed in a thermocycler at 37 ^o^C for 5 minutes and the temperature was slowly decreased by 1 ^o^C per 30 seconds to a final temperature of 4 ^o^C. The cells were then analyzed using flow cytometry (BD FACSCantoTM II and BD FACSAriaTM III), keeping settings constant. Flow cytometric data were assessed using FlowJo, and mean fluorescence intensities were evaluated for each fluorescent color. All cells used were purchased from ATCC.
6. *Transmission electron microscopic (TEM) analysis of cellular uptake of cancer diagnocodes*: After three minutes of trypsin treatment, 1 × 10^7^ cells were collected and transferred to a 1.5-ml test tube, washed three times with PBS, and suspended in 1 ml of complete media (RPMI 1640 supplemented with 10% FBS). The cells were fixed with 2% paraformaldehyde and 2% glutaraldehyde (0.05 M sodium cacodylate buffer, pH 7.2) for 2 hrs at 4 ^o^C and washed three times with 0.05 M sodium cacodylate buffer (pH 7.2). Next, the cells were post-fixed with 1% osmium tetroxide (0.05 M sodium cacodylate buffer, pH 7.2) for 2 hrs at 4 ^o^C, washed twice with distilled water at room temperature, and then bloc-stained with 0.5% uranyl acetate overnight at 4 ^o^C . The cells were then sequentially dehydrated with 30, 50, 70, 80, 90, 100, 100 and then 100% ethanol, each for 10 minutes at room temperature, followed by incubation with 100% propylene oxide for 10 minutes at room temperature twice. Finally, the cells were infiltrated with a 1:1 mixture of propylene oxide:Spurr’s resin for 2 hrs followed by a 0:1 ratio for 4 hrs and a 0:1 ratio for 2 hrs. The resin was polymerized at 70 ^o^C for 24 hrs. The resulting sample was sectioned with an ultramicrotome (MT-X, RMC, Tucson, AZ, USA), stained with 2% uranyl acetate for 7 minutes and then with Reynolds’ lead citrate for 7 minutes, and observed with TEM (JEM-1010, JEOL).
7. *Quantitative Real-Time Polymerase Chain Reaction (qRT-PCR)*: qRT-PCR was conducted as previously described^[2]^ after 3 days of culture. Total RNA from the cells (n=3 per group) was prepared using an RNeasy Mini kit (Qiagen, Chatsworth, CA, USA) according to the manufacturer’s instructions. RNA concentration was determined by measuring the absorbance of the samples at 260 nm using a spectrophotometer (Nanodrop ND-1000, Thermo Scientific, Waltham, MA, USA). cDNA was prepared by reverse transcription of RNA samples with a Takara PrimeScript II First Strand cDNA Synthesis Kit (Takara, Shiga, Japan) using 5 ng of pure total RNA. qRT-PCR was conducted using TaqMan Fast Universal PCR Master Mix (Applied Biosystems) with a StepOnePlus Real-Time PCR System (Applied Biosystems, Foster City, CA, USA). The gene expression profiles were quantified with TaqMan Gene Expression Assays (Applied Biosystems) for each target (EZH2; Hs00544833_m1, MIR21; Hs04231424_s1, and glyceraldehyde 3-phosphate dehydrogenase (GAPDH); Hs02758991_g1). The expression of each target gene was quantified using the comparative Ct method, which normalizes the expression to an endogenous reference (GAPDH).

**Suppplementary References**

[1] K. S. Park, S .W. Shin, J-H. Choi, B-K. Oh, J-W. Choi, S. H. Um, *Biotechnol. Bioprocess Eng.* **19(2)**, 262 (2014).

[2] S. Han, K. Yang, Y. Shin, J. S. Lee, R. D. Kamm, S. Chung, S.-W. Choi, *Lab Chip.* **12(13)**, 2305 (2012).
